# Collectanea

**Published:** 1827-02

**Authors:** 


### COLLECTANEA.

Floriferis ut apes in saltibus omnia libant,
Omnia nos, itidem, depascimur aurea dicta.

### ANATOMY.

*Case in which the Parietal Bones were divided into two, on either side, by a longitudinal Suture.*—The cranium appeared to be that of a person between thirty and fifty years of age. The suture dividing the parietals was dentated, and had several ossa wormiana. The mastoid apophyses of the temporal bones were likewise separated from the rest of the bone by a suture. This formation was evidently congenital. (*Zeitschrift für Physiologie.*)

*Two new Articulations in the Vertebral Column.*—Professor Mayer, of Bonn, has recently published a Memoir, (see the German work above quoted,) in which he has described two new articulations in the spinal columns of adults, who have possessed great freedom of motion in the back. The first of these is found between the spinous apophyses of the lumbar vertebræ. The inferior border of the spine of one vertebra unites by a small articular condyle with a corresponding facette, or articular cavity, on the upper border of the spinous process next in succession. The author calls this joint the interspinous diarthrosis of the lumbar vertebræ. It is said to be most distinct between the third and fourth of these, in which situation a distinct synovial capsule is stated to exist.

The second articulation, less common than the preceding, is formed from the ninth dorsal to the second lumbar vertebræ. It is constituted by two facettes and two articular condyles, at each of the above-mentioned vertebræ. The facettes are situated beneath and within the superior oblique apophyses at the upper border of the root of the spinous processes; the condyles are placed at the corresponding part of the inferior surface of the vertebra. M. Mayer calls this the oblique accessory diarthrosis; the facettes, the accessory glenoid cavities; and the articular condyles, the oblique accessory apophyses. (*Ibid.*)

### PHYSIOLOGY.

*On the Luminousness observed in the Eyes of various Animals.*—The following observations are from a paper by Dr. Charles L. Esser, in Professor Jameson's Journal for January.*

Appearances of light, as is well known, are not uncommon in inferior animals, and the number of luminous animals in the sea are so great, that large tracts of the water's surface have been seen to be illuminated by them.

This phenomenon, however, is comparatively seldom observed in fishes, and the

* Translated from Karsten's Archiv. b. viii. heft iv.

more rarely the higher we ascend in the scale of the animal kingdom, if, under the denomination of luminousness, we understand the real evolution of light, and do not consider it as the reflection of the incident rays of light; for, in this latter case, the luminous appearance does not inhere in the animal body itself, but is in reality merely a reflection, which is totally different from the evolution of light in the inferior animals. A real phosphorescence is sometimes observed in the higher animals, and even in human beings, particularly in their excrementitious fluids. The light of the eggs of the lizard, the luminousness of the perspired matter in man and animals, the irradiation of light in cats and other animals, from the stroking of their hair,—and, finally, the phosphorescent quality of human urine, have been frequently observed.

On most of these various kinds of light, I have neither performed experiments myself, nor have I collected the facts of others; the present memoir being chiefly devoted to an examination of the light or luminousness of the eyes in human beings and inferior animals.

The more perfectly to accomplish this object, I some years ago performed a series of experiments, that led to an important result.

Having brought a cat into a room half darkened, I observed that the eyes of the animal, when opposite the window, and in a certain direction to myself, sparkled very brilliantly; which phenomenon suddenly vanished, when I, either by the motion of my head, changed the direction of my eyes to those of the cat, or the animal, by moving its eyes to and fro, brought them into a different position. In a situation wherein I could best observe the eyes of my cat, I caused the room to be slowly darkened, by gradually closing the window-shutters. The light of its eyes became weaker, and vanished entirely as soon as the room, on the place where the cat was situated, became absolutely dark. Incident rays of light were always necessary to produce the luminousness of the eyes.

I wrapped another cat in a cloth, but left the head uncovered, whereby I was able to handle the animal as I had a mind, and place it in any situation I chose. In this cat what I have just stated was confirmed. I placed it in such a position that its head, at the distance of a few steps, was directed towards the window, by which means I could lighten or darken the room at pleasure. I now permitted a few rays of light to fall through the window into the room, in such a manner that the place where the cat was present was illuminated; and I placed myself in such a direction towards the window, that my eyes were in a straight line with those of the animal, so that I saw the light of its eyes very distinctly; which light, as in the former experiment, suddenly vanished when I turned my head, or the cat turned its eyes. At the moment when my eyes were directed in the manner just mentioned, I observed a most beautiful green light; but, when they were out of this direction, the cat's eyes had their usual appearance. By the turning of my head, or by any other arrangement I chose, by which I intercepted the incident rays, I could at pleasure cause sometimes the one eye of the cat, sometimes the other, and sometimes both together, to shine. If I intercepted the incident rays of light from the left half of the head, the right eye became luminous, and conversely. In these experiments, I observed quite distinctly that the light of my cat's eye emanated from the pupil, the eye itself being lightened only in proportion to the dilatation of that part of it. By suddenly admitting a strong glare of light into the room, I produced a contraction of the pupil; and, when I suddenly rendered the room somewhat dark, a small round luminous point first appeared in the eye, and that point enlarged according as the pupil was dilated. The pupil of the eye of these animals being thus dilated in imperfect darkness, so that the iris seems to encircle the pupil as a small ring, and the sclerotic in cats being scarcely visible, may be the reason why it is believed that the whole eye of the cat is luminous, although its light is, nevertheless, only in proportion to the dilatation of the pupil.

The dilatation of the pupil in twilight is, however, not the only cause of the light of the eyes; but the light surrounding the animal being fainter, also assists us in perceiving with greater distinctness the light as it is more vividly reflected from their eyes; for, if we suddenly illuminate a chamber in which there is a cat, there remains nothing but a luminous brightness where there was formerly a beautiful yellowish green light.

The light of my cat's eyes appeared to be more vivid when she opened them wide from apprehension, or looked around her attentively; whence Treviranus observed, that the eyes of cats sparkled most when the animals were in a lurking position, or in a state of irritation. That author says—

"The light of the cat's eyes appears most conspicuous when she is in a lurking position,—when she is attracted by any unusual appearance,—or when irritated. In the first two instances, the light is faint and dull: in the last instances, it darts forth in intermitting scintillations, and at those movements when the light is most vivid, there are accompanying movements of the eyes." That the light of the eyes of animals appears brighter in a state of irritation than in a state of quiescence, seems to originate in this, that the eyes of all animals, as well as those of man, appear brighter in violent rage, and sparkle more, than in a quiescent state. This, in man, seems to arise from an increased secretion of the lachrymal fluid on the surface of the eye, by which fluid the light of the eye is undoubtedly rendered more brilliant. Treviranus further observes, "The eyes of the cat shine also where no rays of light penetrate, and the light must in many, if not all, cases proceed from the eye itself." Before performing the above experiments, I entertained the same opinion with Treviranus, and made many fruitless experiments with cats in the dark, before I abandoned the position. The light must be brighter in proportion to the darkness of the place where the cat is. I soon renounced this opinion, when, in all the experiments I made on cats in places absolutely dark, I did not discover the slightest trace of light in the eyes of these animals, let me irritate them as I could.

Besides cats, many domestic, as well as other animals, are furnished with luminous eyes.

Under similar circumstances as above, I observed that the light of a dog's eye, as was the case in my experiments on cats, vanished suddenly as soon as I had completely darkened the room where the dog was. I observed the eyes of another dog sparkle when he was irritated, and in the corner of a room that was faintly lighted. The eyes of the animal protruded very much, glittered brilliantly, and the pupils were dilated to an unusual degree. The colour of the light, which was commonly yellow, changed more or less as the rays of light fell on the eyes of the animal, and exhibited the following appearances:—When a small body of rays of light fell on the eye, the light was of a fiery redness, and sometimes so strong that, after I looked a long time attentively at it, my own eyes experienced a disagreeable sensation. When there was a great body of rays, the light was green or yellow, sometimes bluish. In respect to this change in the colour of the light, I was inclined to think that it might be owing as much to the motions of the animal's eyes as to the body of light that fell upon them. This change was different in different dogs, and in some it was not at all observable. Further, the eyes of every dog placed in the same situation shone, but the intensity of the light varied with the individuals.

I have observed luminousness in the eyes of horses, sheep, and hares, which was different, however, in colour and strength.

Many appearances of light have been observed in the eyes of human beings. Treviranus mentions that G. T. L. Sachs, and his sister, both belonging to Albinoes, had phosphorescent eyes. Late in the evening there appeared in them a lively yellowish brightness, which darted forth in fiery coruscations or globules from the interior of the eyes. The balls rolled hither and thither, and frequently ejected rays at least an inch in length. In these two relatives the light was liveliest and strongest after their birth, and during infancy; in their more advanced years, the light was strongest when they were in deep meditation: at this time also the oscillation, which they had in common with other Albinoes, was liveliest.

A rather remarkable observation, and similar to the case of the Sachs, is that of Michaelis, who, many years before his death, during the interval between day and night, and during the night itself, observed irradiations of light issuing from his eyes,—sometimes so strong that he could read the smallest print. (SCHLICHTEGROLL's Necrolog, des 19 Jahrhunderts, B. 3. s. 337.)

In a boy, who belonged to the Albino variety, I observed a similar case, though not accompanied with irradiation. In this boy, who suffered so much from the dread of light that he never ventured abroad except in twilight, I frequently observed the same fiery eyes; yet were they very different, both in the strength and colour of their light, from the luminous eyes of animals which I had observed, partly from design and partly from accident, for this boy's eyes might be called glassy rather than luminous. Some years ago I was assured, by Hr[n] Geheimenrath W., that his sister had often observed the eyes of her children, who were also Albinoes, to be luminous.—These last two cases could be traced to rays of light falling on the eyes.

It now remained for me to search out the cause which, by means of the incident rays of light, gave rise to the shining appearance in the eyes of human beings and inferior animals. This explanation seemed to me no easy matter, yet, from the beginning, I expected to be able to search out the cause of this phenomenon in a reflection of rays of light penetrating into the eye. The colour of the light, however, and particularly its changes in dogs, appeared to me very difficult to explain, and to be rather at variance with my own opinion.

To discover the cause of the shining in the eyes of human beings and inferior animals, I came to the resolution of undertaking the extraction of the lens on a cat, from which I anticipated the best result, in so far as I might, by that means, best determine to what extent the remoter parts of the eye contributed to its luminousness.

I attempted to perform the above operation on a cat, but the utter restlessness of the animal rendered it extremely difficult,—indeed, almost impossible. Having ascertained that eyes of cats shine after death, I resolved to kill the cat, that I [illegible]ght have it in my power to dissect any part of the eye I thought proper.

First, by means of a pair of scissors, I cut away the whole of the cornea, and completely destroyed the anterior chamber of the eye. I now observed that the light of the eye was not in the least diminished, but somewhat weakened in regard to colour, which was changed from a yellow to a pale green. I then took away the iris, that lay exposed before me, without injuring the conformity of the hinder part of the eye, to disc[illegible] whether the iris, as Treviranus maintained, really contributed to the light. This, however, was not the case; for the light still continued. The taking away of the lens was followed by a different result, which considerably weakened the intensity of the light, and the greenness of its colour. It now struck me that the tapetum in the hinder part of the eye must form a spot, which caused the reflection of the incident rays of light, and thus produced the shining. This was the more probable, as the light of the eye now seemed to emanate from a single spot. After taking away the vitreous humour, I observed that, in reality, the entire want of the pigment in the hinder part of the choroid coat, where the optic nerve enters, formed a greenish silver-coloured changeable oblong spot, which was not symmetrical, but surrounded the [illegible] in such a manner that the greater part was above, and only a small [illegible]ow it; and therefore the greater part lay beyond the axis of vision. It is this spot, therefore, that produces the reflection of the incident rays of light, and, beyond all doubt, according to its tint, contributes to the different colouring of the light; to which, nevertheless, the remaining parts of the eye, when conjoined, seems to be no less necessary.

*Time required by different Substances to manifest their Presence in the Urine.*—Some curious experiments have recently been made by M. G. A. Stebberger, with a view of ascertaining the periods which intervened between the introduction of different substances into the body, and their being detected in the urine.

A young man was affected with a congenital prolapsus of the bladder. This malformation presented a red fungous excrescence at the lower part of the abdomen, which was always moist, and very sensible to the touch. The urine constantly oozed from the orifices of the ureters, which might be seen, by which the fluid might be collected in the state it was secreted by the kidneys. When the urine was examined in its natural state, and while the young man fasted, it was found to be of a deep yellow colour, with a nauseous odour and salt taste: it converted into blue tournsol paper, which had been reddened by an acid. A certain quantity of it was always collected in the first instance before beginning an experiment, and this was compared with what was secreted during the continuance of the operation. When any substance was exhibited, the urine was collected every ten minutes, till the presence of the matter in question was perceived. Intervals of a quarter of an hour, or half an hour, were then interposed until the urine had regained its natural state. In this way the progressive increase and diminution of the quantity of foreign matter in the secretion was readily observed.

1. The following substances, introduced by the mouth, *were* detected in the urine:—The colouring matter of rhubarb, that of black cherries, that of madder, of logwood, of indigo, of the pulp of the cassia, gallic acid, the tannin of uva ursi, hydrocyanates of potass and of iron, and one of the constituents of elder; which last gave to the urine a deep yellow colour.

2. The following matters, when introduced by the mouth, *were not* detected in the urine:—Tincture of tournsol, (according to the experiments of Tiedemann and Gmelin, this matter is destroyed in the alimentary canal,) the bitter principle of quassia, the tincture of iron, and the acetate of iron.

3. Substances employed in the form of baths and fomentations, or embrocations, were not perceived in the urine, with the exception of oil of turpentine and acetate of potass: the former, indeed, is well known to communicate a violet odour to the urine by simply inhaling the vapour. The other substances tried were infusion and tincture of rhubarb, decoction of logwood, and a solution of gallic acid. (*Zeitschrift für Physiologie.*)

---

### PATHOLOGY.

*Successful Inoculation of the Measles,* from the Edinburgh Med. and Surg. Journal, January.)—

This inoculation, which was performed with success by Home and Horst, and recommended by Vogel, Percival, Brown, Monro, and Tissot, but was afterwards condemned by Cullen, Girtanner, Rosenstein, Vaidy, and Montfalcon, was again employed with advantage by Professor Speranza, in an epidemic which prevailed in the territory of Mantua in 1822. Six boys in the House of Industry, and afterwards he himself, were inoculated with the most evident effect in propagating the disease, which in all followed a mild and regular course. A repetition of the experiment, by himself and others, had the same fortunate issue. The inoculations were performed in the following manner:—A slight cut was made into one of the most vivid of the large spots with a lancet, the point of which was covered with the blood effused. With this some small incised punctures were made on the arm, and a proper bandage applied. The phenomena of inoculation commonly appeared in a few days. (*Bibliotheca Italiana.*)

### PRACTICAL MEDICINE.

### *Cases of Nervous Irritation, exhibiting the Efficacy of Cold as a Remedy.* By S. Jackson.—

If any diseases may be considered as distinct from all others, with respect to their essential character, they are cramps, convulsions, &c., vaguely classed, in the common nosological systems, with the designation of neuroses. On the principles of the physiological medicine, they are diseases of irritation, of the same general character as other diseases of irritation, and receive their peculiar character, or the phenomena they present, solely from their location in the nervous system. Their treatment is to be directed on the same principles as other diseases of sur-irritation, —that is, by direct debilitants addressed to the primary seat of the irritation, or by revulsives.

The irritation that occasions nervous phenomena may be entirely confined to the nervous fibrils of an organ, in which it commences, and from which it is transmitted to the brain, and thence reflected into the voluntary muscles and other tissues; or it may be complicated with sanguine vascular irritation of the same organ. These circumstances impart a difference to the phenomena attendant on it, and the method of treatment. In the first case, it commonly yields without difficulty to the diffusible stimuli, to narcotics, antispasmodics, and revulsion practised on the exterior. In the last, those remedies are of less assured efficacy, not unfrequently fail entirely to subdue the affection, and sometimes aggravate it. The agents that debilitate the organic actions, that allay irritation, applied directly to the organ where it is seated, in conjunction with revulsives, are the means indicated; and, simple as they may seem, are attended with most prompt and decidedly beneficial effects. The following cases belong to this last class, and are well-marked examples of the morbid condition I have alluded to, as well as of the treatment I consider most appropriate to it.

Case I.—July 23, 1825.—The heat had been excessive for several days; thermometer ranging from 90° to 98° F. I was called to a man supposed to be suffering from having drunk cold water. The subject was about thirty-five years of age, fair complexion, stout built, and nervoso-sanguine temperament. He was an Irishman by birth, and a weaver by profession. He had worked steadily during the day at his loom, in a confined and very warm room; had been very thirsty, and drunk largely of spirits and water, but not sufficient to intoxicate him. In the evening, he walked out, after eating heartily, and on his return was suddenly seized with giddiness, and inability to stand. He was carried home, and, from a supposition that his disorder had been induced by cold water, spirits and laudanum were given to him. The symptoms were immediately aggravated, and, in a few moments after, were followed by violent spasmodic and convuls[illegible] efforts.

In this state I saw him. It was with difficulty four or five athletic men could retain him on a bed. The face was flushed, distorted with an expression of anguish; the eyes fiery. The convulsive throes came on in paroxysms, which lasted five to six minutes, and with short intervals; in the intervals, jactitation, tossing of the arms, cries of anguish; pulse was frequent, full, and oppressed; skin hot; profuse sweat covered the face and neck; epigastrium exceedingly sensitive, pressure on it raised loud complaints, and renewed the convulsive exertions; thirst intense. Consciousness was perfect, but the mind, concentrated on the sufferings experienced, could not be brought to attend to any inquiries addressed to the patient.

The diagnosis formed of the case was—vascular and nervous irritation of the stomach; the predisposition to gastric irritation, derived from the extreme heat; the irritation itself excited by the use of ardent spirits during the day, and meal in the evening, suddenly aggravated by the spirits and laudanum, administered as remedies; excitement of the general vascular system, and irritation of the portion of the cerebral structure presiding over the voluntary movements, transmitted sympathetical from the stomach.

The treatment was directed by this view. A tub of cold water from a well-pump was ordered, and a vein opened. While the blood was flowing, a stream of cold water was directed to the head, and cold water given in small draughts. At the commencement of the treatment, a convulsive paroxysm came on: it soon ceased, and proved to be the last. Twenty ounces of blood subdued the vascular excitement. The cold drinks and affusion were in the highest degree grateful, and called forth, from the patient, the most extravagant expressions of the relief they afforded him. He now informed me that the head and stomach were the seats of the anguish he experienced, and that, although he had been conscious of what he was doing, he could not control or restrain the violence of his muscular exertions.

Cloths dipped in cold water were applied to the epigastrium; iced gum water, acidulated with lemon-juice, was directed to be given during the night; and a domestic clyster to open the bowels.

July 24.—No return of convulsion; violent pain in stomach and bowels, attended with copious discharge of blood; pulse full and tense.—V.S. ℥ xij. Injection of cold water. Sal Rochelle ʒ j. dissolved in a pint of water, wine-glassful every hour. Continue gum water.

July 25.—Pain removed; discharge of blood per anum ceased after the first injection of cold water; skin soft and cool; pulse natural; tongue furred.—Continue gum water.

July 26.—Convalescent.

Case II.—January 3, 1826.—Called to see a man in the employ of a livery-stable keeper, as an hostler; age, twenty-five; fair complexion, light hair and eyes; slight figure; a Swiss by birth; sanguine nervous temperament.

In the evening, immediately after having eaten supper, he had been seized with great distress, attended with violent convulsive efforts. I found him on the floor, struggling with several persons, who held him down. He uttered cries of anguish, seemed in great torture, was perfectly conscious, but could not express his feelings. When interrogated, he pointed to his stomach as the seat of pain; the tongue scarlet red on the edges, and furred; the skin was cool, and pulse feeble. I was informed by his employer, an Irishman, that he had been very thirsty for several days, and had drunk large quantities of cold water, to which he attributed the present condition of the patient; and was very pressing with him to drink some whiskey, but which was rejected with an expression of horror. I also learnt he had dined that day on salt pork and cabbage.

To a pitcher of cold pump water was added four ounces of sugar, and the patient was directed to drink of it, in small quantities, every few minutes. It was swallowed with the greatest avidity, and, had he been permitted, would have gorged himself with it immediately. Relief was almost instantaneous after the first draughts. The convulsive efforts ceased; the patient sat up, and could describe his sensations: he felt as if fire was in his stomach. A pediluvium was ordered, with warm fomentations to the abdomen, frictions to the extremities, and an injection into the rectum. On my return in half an hour, all the accidents were dissipated; the patient was sitting up, and in comparative ease. He had finished his pitcher of drink, and had commenced upon another. Vascular excitement had come on; ten ounces of blood were drawn; the cold drink continued. Next morning, the patient was attending to his duties; and a regimen for a few days entirely restored the stomach to a healthy condition.

### The following case is related by Dr. La Roche.

Mrs. F—, about thirty years of age, of a sanguine and nervous temperament, was attacked about four years ago, whilst residing in the State of Alabama, with a violent pain in the epigastric region, attended with vomiting. It occurred soon after dinner, and was probably caused by something she had eaten. No physician being at hand, her husband gave her tea-spoonful doses of laudanum and chamomile tea; which, however, were ejected from the stomach with considerable efforts, and an aggravation of the symptoms. The gastric irritation and pain soon became so violent as to occasion severe convulsive movements in almost every muscle of her body, and to deprive her of her senses for more than six hours. From this very severe attack she, however, recovered, more by chance and through the efforts of her good constitution, than from the effects of medicine. Since that period she has continued subject to this complaint. The attacks are more or less severe, are brought on by the slightest irregularities in regimen, and are in general with difficulty removed.

At two o'clock on the morning of the 23d of April last, she was once more attacked with this painful complaint, and suffered severely until eight o'clock, when I was requested to visit her. I learned from her friends that she had been slightly indisposed for a few days, and had eaten the preceding evening a small portion of lobster. This, together with the greater part of what she had eaten during the day, had been ejected from the stomach a short time previous to my visit. I was also informed that she had taken twenty drops of laudanum, warm teas, and that warm flannels had been applied to the region of the stomach. Her pain was now hardly to be endured; the muscles of her upper extremities, as well as of her neck and face, were spasmodically contracted ; her skin was covered with cold perspiration ; and her pulse, in the short intervals of the convulsions, was found to be greatly accelerated. Judging, from the severity of these symptoms, that no time was to be lost, and influenced by former prejudices, I immediately directed forty drops of laudanum to be administered in combination with a little of the essence of peppermint, (Mrs. F— never taking laudanum without it,) and a perseverance in the warm tea, &c. A short time after the exhibition of the laudanum, the pain was aggravated, but was soon a little relieved, in consequence of vomiting supervening. Another dose was soon administered, which again occasioned an increase of the symptoms, and brought on vomiting, by which the stomach was completely cleared. A mustard poultice was ordered to be applied to the epigastric region ; but, as it required some time to be prepared, I judged it adviseable to resort, in the mean time, to something, to lessen, if possible, the sufferings of the patient. As laudanum, and other remedies usually applied in such cases, instead of abating, seemed to aggravate the pain, I determined to give a trial to cold water, as prescribed in nearly similar cases by my friend Dr. Jackson, of this city. A tumblerful of very cold spring water was in consequence procured, one-half of which the patient was requested to take immediately. In less than three minutes, some relief was obtained. An equal quantity of the water was given with a still greater, and indeed a remarkable, mitigation of the pain.

The poultice was now applied, and was about ten minutes before producing an inflammation in the skin. During the time, however, Mrs. F— had drunk a second tumbler of the water, had slept a few minutes, remained free from spasms in the muscles, and felt completely relieved from the pain. The poultice was taken off about fifteen minutes after its application, and the patient directed to drink frequently through the day of water sweetened with orange-flower syrup. In the afternoon, she experienced some slight spasmodic pains in the stomach, in consequence of eating some thin sago, but found relief in a draught of her water. The tongue, however, remained red and somewhat parched ; the pulse was quick ; skin a little hot ; the head painful, and the thirst considerable. The water was directed to be continued, and an emollient injection ordered, in order to relieve a sense of weight and uneasiness in the bowels. The next day, I was very happy to find that all signs of gastric irritation had subsided. The bowels being costive, and the tongue a little foul, but pale, a dose of Epsom salts and calcined magnesia was prescribed, and served to remove every vestige of the complaint. With the exception of the irritation caused by the mustard, Mrs. F— was immediately restored to perfect health.

(*North American Medical and Surgical Journal.*)

---

### MATERIA MEDICA.

*On the Diuretic Properties of the Equisetum,* (from the Edinburgh Med. and Surg. Journal, January.)—

The various species of the Equisetum have been recommended by Professor LENHOSSEK, of Vienna, as a very powerful and specific diuretic, which neither oppresses the digestive organs, nor induces any bad consequences in the vascular or nervous systems, and is therefore preferable to squill, digitalis, colchicum, and other diuretic remedies, whose unpleasant consequences are too well known. It is particularly serviceable in serous accumulations from debility, or after exanthematic fevers, and is contraindicated in inflammatory states of the system. All the species of Equisetum possess a directly diuretic virtue: the Arvense, Variegatum, Ramosum, and Palustra, act more mildly; but the Hyemale and Limosum act more powerfully, and are more apt to induce bloody urine. The dry plant is also preferable to the recent, which is more active. It has been sometimes given in powder, but the decoction answers well in every case, as it is not offensive either in smell or taste, and children take it readily when sweetened. Two or three drachms of the dried herb are to be boiled in a pint of spring or river water for a quarter of an hour, and of the decoction a spoonful or two may be given to children, according to their age, and adults may take half a cupful, or a whole cupful, every two hours, until the flow of urine be increased. (*Beobachtungen und Adhandlungen von Oesterreicheschen Aerzten,* vol. v.)

